# Saline-Alkaline Stress Suppresses Soybean Germination and Early Seedling Growth via Induction of DNA Damage in Roots

**DOI:** 10.3390/plants15071131

**Published:** 2026-04-07

**Authors:** Gege Yang, Rui Sun, Yingyi Zhang, Jiaxin Song, Jiahui Li, Zhihui Luan, Wenjing Qi

**Affiliations:** Department of Bioscience, Changchun Normal University, Changji North Road 677, Changchun 130032, China

**Keywords:** saline-alkaline stress, (*Glycine max* (L.) Merr.) germination, DNA damage response, antioxidant response

## Abstract

Saline-alkaline (SA) soils pose a serious threat to soybean production worldwide. Although severe saline-alkaline stress can reduce yield by up to 30%, the mechanisms underlying saline-alkaline-induced inhibition of root growth remain unclear. In this study, two soybean cultivars with contrasting tolerance, Chang Nong 26 (CN26) and Jiyu 441 (JY441), were exposed to saline-alkaline stress induced by NaHCO_3_ and Na_2_CO_3_ at Na^+^ concentrations of 0, 21, and 45 mmol·L^−1^. The effects on seed germination, early seedling growth, antioxidant responses, and root DNA damage were systematically examined. High-level saline-alkaline stress significantly inhibited germination and root elongation in both cultivars. Superoxide dismutase (SOD) and peroxidase (POD) activities increased markedly under stress, indicating activation of antioxidant defenses. Catalase (CAT) and ascorbate peroxidase (APX) to scavenge ROS and maintain cellular redox balance. Nevertheless, oxygen-free radicals (OFRs) accumulated to a significantly greater extent in the root tips of CN 26 than in JY441, suggesting lower tolerance in CN 26. Random amplified polymorphic DNA (RAPD) analysis revealed pronounced DNA damage in root tips under saline-alkaline stress, with more polymorphic bands detected in CN 26 than in JY441. Furthermore, qRT-PCR analysis demonstrated that the expression of DNA damage repair-related genes (*RAD51*, *OGG1*, *RAD4*, and *ATM*) was downregulated in CN 26 roots under stress, whereas *E2FA* and *WEE1* expression was upregulated. In contrast, these DNA repair genes in JY441 were significantly induced during the early stage of stress exposure and subsequently declined. Collectively, this study demonstrates that saline-alkaline stress inhibits soybean growth through the induction of oxidative DNA damage and cell cycle arrest in roots. The reduced capacity for DNA repair in CN 26 likely contributes to its greater sensitivity to saline-alkaline stress. This study provides mechanistic insights into saline-alkaline stress-induced growth inhibition in soybean and offers a theoretical basis for breeding stress-tolerant cultivars.

## 1. Introduction

Soil salinization significantly impacts plant growth, crop productivity, and soil efficiency, leading to a range of ecological and environmental issues [1,2]. Saline soils are typically composed of a mixture of neutral and alkaline salts [3,4], and in arid and semi-arid regions, both saline and alkaline conditions often co-occur, subjecting plants to combined saline-alkaline stress. In such environments, plants face not only high ion concentrations but also elevated pH caused by CO_3_^2−^ and HCO_3_^−^ [5,6,7]. In Northeast China, a major soybean-producing region, saline-alkaline soils are predominantly characterized by high concentrations of Na_2_CO_3_ and NaHCO_3_, with pH values often exceeding 9.0 [8]. The presence of Na^+^ in NaHCO_3_ and Na_2_CO_3_dominated soils induces ionic toxicity, while HCO_3_^−^ and CO_3_^2−^ elevate soil pH, resulting in combined ionic and alkaline toxicity [9,10]. Saline-alkaline (SA) soils adversely affect seed germination and seedling growth, impacting multiple plant organs’ development [11]. Furthermore, they inhibit critical physiological processes, including photosynthesis, protein synthesis, energy and lipid metabolism, and respiration, ultimately leading to reduced yields or crop failure [12,13]. High pH caused by alkalinity directly targets roots, disrupting root growth and cell differentiation, altering cellular structures, and impairing membrane stability, which in turn affects transmembrane potential and causes metabolic dysfunction [14].

Saline-alkaline stress induces excessive reactive oxygen species (ROS) production in plant cells. ROS can damage DNA, resulting in various forms of DNA damage, such as chromosomal breakage and base mutations [15,16,17]. Numerous studies have demonstrated that oxidative DNA damage can trigger programmed cell death, inhibit plant growth, accelerate senescence, or even cause organismal death in plants, bacteria, and animals [18,19,20]. To counteract ROS overaccumulation, plants activate antioxidant enzymes such as superoxide dismutase (SOD), peroxidase (POD), catalase (CAT), and ascorbate peroxidase (APX) to scavenge ROS and maintain cellular redox balance [21]. Studies have shown that plant antioxidant enzyme activity is influenced by stress intensity, and that moderate stress can enhance antioxidant activity, while higher stress levels may lead to reduced enzyme efficiency [4,22,23,24,25,26]. Additionally, DNA damage repair (DDR) mechanisms such as mismatch repair (MMR), base excision repair (BER), nucleotide excision repair (NER), non-homologous end joining (NHEJ), and homologous recombination (HR) play a critical role in mitigating ROS-induced DNA damage [27,28,29,30]. Cell cycle checkpoints can perceive DNA damage signals and subsequently inhibit or arrest cell cycle progression, allowing sufficient time for DNA repair [31].

Soybean (*Glycine max* (L.) Merr.) is a major global source of plant protein and oil and an important feed crop. Although soybean is considered moderately salt-tolerant, excessive salinity and alkalinity can significantly inhibit seed germination and plant growth, suppress root nodule formation, reduce yield, and impair seed quality [14,17,32,33]. However, the underlying mechanisms of soybean’s response to SA stress at multiple biological levels remain insufficiently explored. This study aims to investigate the effects of NaHCO_3_- and Na_2_CO_3_-induced stress on soybean germination, seedling growth, antioxidant responses, and DNA damage. Two soybean cultivars, Jiyu 441 (JY441) and Chang Nong 26 (CN26), with differing sensitivities to saline-alkaline stress, were analyzed. Seed germination, root growth, antioxidant enzyme activities, ROS metabolism, and genomic DNA damage were assessed to clarify the mechanisms underlying the effects of saline-alkaline stress on soybean. This study provides important insights for breeding salt- and alkaline-tolerant soybean varieties and enhancing seedling establishment in saline-alkaline environments.

## 2. Materials and Methods

### 2.1. Materials and Treatment Conditions

Soybean (*Glycine max* (L.) Merr.) seeds of two cultivars with contrasting saline-alkaline (SA) tolerance, JY441 (an alkaline-tolerant cultivar) and CN26 (an alkaline-sensitive cultivar), were provided by the Jilin Academy of Agricultural Sciences (Jilin, China). Uniform, healthy seeds without visible damage were selected and surface-sterilized in 0.5% (*v*/*v*) NaClO solution for 5 min, followed by three rinses with tap water and three rinses with deionized water to remove residual disinfectant. The seeds were air-dried for use. To simulate alkaline soil conditions, treatment solutions were prepared by mixing Na_2_CO_3_ and NaHCO_3_ to obtain Na^+^ concentrations of 21 (pH 8.92) and 45 mmol·L^−1^ (pH 9.86) [34]. The control (CK) received an equal volume of distilled water. For germination assays, the seeds were evenly placed in Petri dishes lined with sterile germination paper and supplied with the respective treatment solutions to maintain adequate moisture throughout the experiment. The soybean seeds were grown in a controlled environment chamber with a light regime of 16/8 h (light/dark) and relative humidity of 50–55% at 28 ± 2 °C for 7 days. Uniform seedlings were selected for further analyses. Three soybean seedlings were selected in each pot. Each treatment included six independent replicates. Three replicates were used for biomass and morphological measurements, and three for physiological and molecular analyses. Root tissues were harvested after 7 days of treatment, immediately frozen in liquid nitrogen for 5 min, and stored at −80 °C.

### 2.2. Biomass and Morphological Measurements

Seed germination and morphological measurements were conducted following established protocols. The number of germinated seeds was recorded daily for six consecutive days. Germination vigor was calculated on day 3, and final germination rate was determined on day 6 [35]. At the end of the experiment, root length and fresh weight were measured according to the methods described by [31].

The following formulas were used:

Germination rate (%) = (Number of germinated seeds/Total number of tested seeds) × 100

Germination vigor (%) = (Number of normally germinated seeds on day 3/Total number of seeds under the corresponding treatment) × 100

### 2.3. Measurement of Antioxidant Enzyme Activities and ROS Content

Frozen root samples stored at −80 °C were used to determine antioxidant enzyme activities and reactive oxygen species (ROS) levels. The activities of superoxide dismutase (SOD) and peroxidase (POD), as well as the content of superoxide anion (O_2_^−^), were measured according to the manufacturer’s protocols (Michybio, Suzhou, China). The assay principles were based on established methods: SOD activity was determined by monitoring the inhibition of nitroblue tetrazolium (NBT) photochemical reduction [36]; POD activity was assayed by measuring the oxidation of guaiacol at 470 nm [37]; and superoxide anion content was determined using the hydroxylamine oxidation method [38] and hydrogen peroxide (H_2_O_2_) content was determined using the titanium tetrachloride method [39].

### 2.4. Estimation of AP-Site Level in DNA

AP-site levels in DNA were quantified using the OxiSelect™ Oxidative DNA Damage AP Sites Quantitation Kit (STA-324, BioCells, San Diego, CA, USA). Total genomic DNA was diluted to 100 ng·μL^−^^1^ in TE buffer. DNA (5 μL) was mixed with 5 μL ARP solution and incubated at 37 °C for 5 min. Subsequently, 90 μL TE buffer, 1 μL glycogen, 10 μL sodium acetate, and 300 μL absolute ethanol were added. The mixture was incubated at −20 °C for 30 min and centrifuged at 14,000× *g* for 20 min. The DNA pellet was washed three times with 70% ethanol, dissolved in 10–20 μL TE buffer (20 ng·μL^−1^), and stored at −20 °C. Subsequent steps followed the manufacturer’s instructions. Absorbance was measured at 450 nm using a Varioskan LUX microplate reader (Thermo Fisher Scientific, Shanghai, China). AP-site density was calculated based on a standard curve generated with the provided DNA standards.

### 2.5. RAPD Analysis

The RAPD reaction mixture (20 μL total volume) consisted of 1 μL diluted genomic DNA (100 ng·μL^−1^), 1 μL random primer (10 mM), 10 μL 2× Taq PCR Mix (CWBIO, Beijing, China), and 8 μL dH_2_O. Eleven random primers were screened for amplification [40]. The random primers are listed in Table S1. PCR amplification was performed under the following conditions: initial denaturation at 94 °C for 2 min; followed by 30 cycles of 94 °C for 30 s, 36 °C for 1 min, and 72 °C for 30 s; with a final extension at 72 °C for 2 min and maintenance at 4 °C. The polymorphism frequency of RAPDs was assessed using 3% (*w*/*v*) agarose gel electrophoresis and calculated as described by Wang et al. [41]. Genome template stability (GTS) was calculated according to Zhao et al. [31] using the formula GTS = (1 − a/*n*) × 100%, where a and n represent the average frequency of RAPD polymorphism in the treated and control roots, respectively. For all the treatments, bands were considered reproducible and were used for polymorphism analysis when detected simultaneously in at least two experimental replicates.

### 2.6. RNA Extraction and qRT-PCR Analysis

Total RNA was extracted from 100 mg frozen root tissue using the Rapid Plant RNA Isolation Kit (Coolaber, Beijing, China) according to the manufacturer’s instructions. RNA quantity and integrity were assessed using a NanoDrop 2000 spectrophotometer (Shanghai, China). First-strand cDNA was synthesized from 1 μg total RNA using the HiFiScript SuperFast gDNA Removal cDNA Synthesis Kit (CWBIO, Beijing, China) in a 20 μL reaction volume. qRT-PCR was performed using SuperStar Universal SYBR Master Mix (CWBIO) in a 20 μL reaction system, with 1 μL cDNA template. Soybean TUBULIN A (NM_001250372) was used as the internal reference gene. The gene-specific primers are listed in Table S2. Amplification was conducted on a CFX-96 real-time PCR system (Bio-Rad, Shanghai, China) under the following conditions: initial denaturation at 95 °C for 30 s; 45 cycles of 95 °C for 5 s, 60 °C for 25 s; followed by a final extension at 72 °C for 5 min. Relative gene expression levels were calculated using the 2^(−ΔΔCt)^ method [42]. Each treatment included three biological replicates, and each biological replicate consisted of three technical replicates.

### 2.7. Statistical Analysis

All statistical analyses were performed using the GraphPad Prism software (version 10.4.1). Data are presented as mean ± standard deviation (SD) of three independent experiments. Differences among treatments within the same cultivar were evaluated by one-way analysis of variance (ANOVA), with significance *p* < 0.05.

## 3. Results

### 3.1. Saline-Alkaline Stress Inhibits Soybean Germination

**Figure 1:**
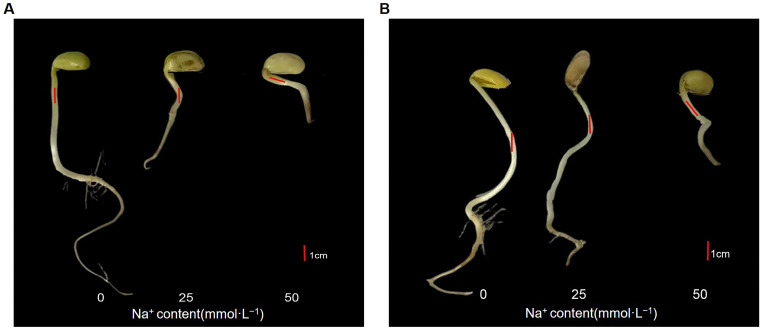
Effects of saline-alkaline stress on soybean germination phenotypes: (**A**) Phenotypic characteristics of CN26 seedlings. (**B**) Phenotypic characteristics of JY441 seedlings. Scale bar = 1 cm.

Based on these previous findings [43,44] and our preliminary experimental results, we selected two Na^+^ concentrations (21 and 45 mmol·L^−1^) of mixed alkaline salts (NaHCO_3_:Na_2_CO_3_ = 1:1) for saline-alkaline stress in this study. To investigate the effects of saline-alkaline stress on soybean germination, the seeds of JY441 and CN26 were treated with mixed NaHCO_3_ and Na_2_CO_3_ solutions containing Na^+^ concentrations of 0, 21, and 45 mmol·L^−1^ for 7 days. Seed germination, seedling morphology, and root morphological characteristics were subsequently examined. Compared with the control (CK), saline-alkaline stress significantly inhibited seed germination in both CN26 and JY441 (Figure 1).

Germination vigor and germination rate are key indicators used to evaluate seed germination performance. As shown in Table 1, the germination vigor and germination rate of CN26 in CK were 0.22 ± 0.11 and 0.34 ± 0.19, respectively, and no significant differences were observed between the saline-alkaline treatments and the control. In contrast, JY441 exhibited higher germination performance under control conditions, with germination vigor and germination rate of 0.98 ± 0.02 and 0.96 ± 0.04, respectively. Saline-alkaline stress significantly reduced both parameters in JY441. Root elongation was also markedly affected by saline-alkaline stress. In CN26, root length under control conditions was 16.30 ± 2.33 cm. Under 21 mmol·L^−1^ Na^+^ treatment, root length decreased to 9.41 ± 1.06 cm (42% reduction), whereas 45 mmol·L^−1^ Na^+^ decreased root length to 3.07 ± 0.17 cm (81% reduction). In JY441, root length was 13.94 ± 3.10 cm under control conditions and declined to 10.08 ± 3.03 cm (27% reduction) and 3.48 ± 0.84 cm (75% reduction) under 21 and 45 mmol·L^−1^ Na^+^ treatments, respectively. These results indicate that high-intensity saline-alkaline stress therefore markedly suppressed root growth in both cultivars.

**Figure 2:**
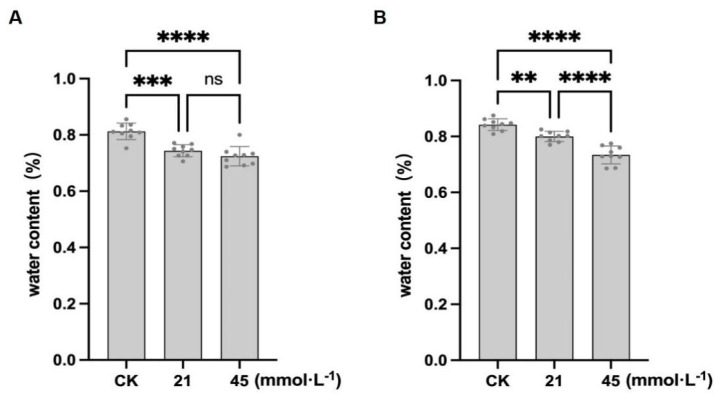
Effects of saline-alkaline stress on water content of soybean seedlings: (**A**) CN26. (**B**) JY441. Data are presented as mean ± SD (n = 3 biological replicates), each replicate consisting of three individual seedlings with three technical replicates. **, ***, and **** indicate significant differences at *p* < 0.01, *p* < 0.001, and *p* < 0.0001, respectively. CK, 0 mmol·L^−1^. ns, not significant.

In addition to inhibiting primary root elongation, saline-alkaline stress also suppressed adventitious root formation in both cultivars, with CN26 showing more pronounced inhibition (Figure 1). Wang et al. [32] reported that alkaline stress significantly reduces adventitious root number and dry weight in soybean. Then, fresh weight (FW), dry weight (DW), and water content were further analyzed to characterize the physiological responses (Table 1). In CN26, FW decreased from 1.06 ± 0.15 g (CK) to 0.79 ± 0.07 g and 0.76 ± 0.07 g under 21 and 45 mmol·L^−1^ Na ^+^, respectively, while DW remained stable (0.20–0.21 g), leading to a decline in the water content from 81.1% to 79.8% and 72.3% (Figure 2A). In JY441, FW decreased from 1.07 ± 0.06 g to 0.75 ± 0.05 g and 0.54 ± 0.04 g, and DW from 0.17 ± 0.02 g to 0.15 ± 0.01 g and 0.14 ± 0.02 g, with the water content declining from 84.1% to 80.0% and 74.1% (Figure 2B), indicating greater water loss than in CN26. Notably, although JY441 exhibited less inhibition of hypocotyl elongation under low stress, its hypocotyl thickness did not increase proportionally. Collectively, these results demonstrate distinct cultivar-specific responses to saline-alkaline stress during germination and early seedling growth.

### 3.2. Effects of Saline-Alkaline Stress on the Antioxidant Defense System in Soybean Seedling Roots

#### 3.2.1. Saline-Alkaline Stress Increases Reactive Oxygen Species (ROS) Accumulation in Soybean Roots

Under saline-alkaline stress, plants produce excessive reactive oxygen species (ROS), including superoxide anions (O_2_^−^) and hydrogen peroxide (H_2_O_2_). To mitigate oxidative damage, plants activate enzymatic antioxidant defense systems that convert O_2_^−^ into H_2_O_2_, which is subsequently decomposed by catalase and ascorbate peroxidase, thereby reducing oxidative injury caused by alkaline stress [32]. In this study, the accumulation of O_2_^−^ in soybean seedling roots under saline-alkaline stress was further examined.

**Figure 3:**
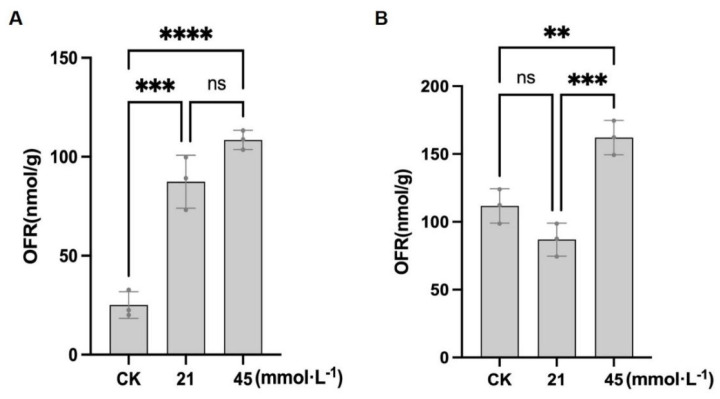
Saline-alkaline stress induces superoxide anion (O_2_^−^) accumulation in soybean seedling roots: (**A**) CN26. (**B**) JY441. Data are presented as mean ± SD (n = 3 biological replicates), each replicate consisting of two individual plants with three technical replicates. **, ***, and **** indicate significant differences at *p* < 0.01, *p* < 0.001, and *p* < 0.0001, respectively. CK, 0 mmol·L^−1^. ns, not significant.

**Figure 4:**
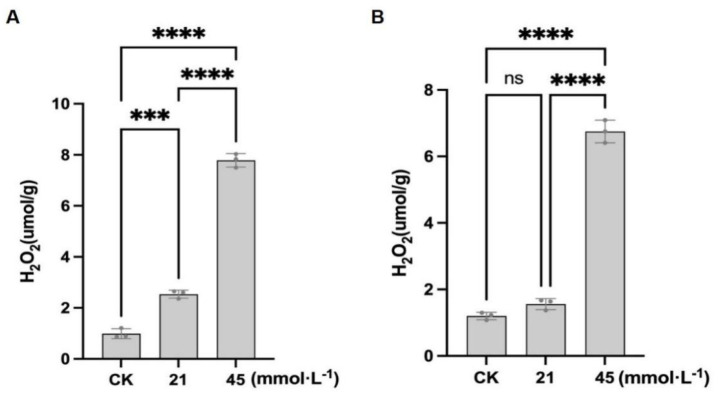
Saline-alkaline stress induces hydrogen peroxide (H_2_O_2_) accumulation in soybean seedling roots: (**A**) CN26. (**B**) JY441. Data are presented as mean ± SD (n = 3 biological replicates), each replicate consisting of two individual plants with three technical replicates. *** and **** indicate significant differences at *p* < 0.001 and *p* < 0.0001, respectively. CK, 0 mmol·L^−1^. ns, not significant.

In CN26, the O_2_^−^ content under control conditions was 25.09 ± 6.75 nmol·g^−1^. Treatment with 21 mmol·L^−1^ Na^+^ significantly increased the O_2_^−^ content to 87.37 ± 13.39 nmol·g^−1^. Under 45 mmol·L^−1^ Na^+^, the O_2_^−^ levels further increased to 108.51 ± 4.85 nmol·g^−1^, representing a 332.5% increase relative to the control (Figure 3A; Table S3). In JY441, the O_2_^−^ content under control conditions was 132.49 ± 26.01 nmol·g^−1^. At 21 mmol·L^−1^ Na^+^, the O_2_^−^ content decreased significantly to 99.07 ± 19.94 nmol·g^−1^ (25.2% reduction), which may be associated with reduced ROS production or a transient enhancement of early antioxidant defense under mild stress conditions. However, under the 45 mmol·L^−1^ Na^+^ treatment, the O_2_^−^ content increased markedly to 200.32 ± 38.08 nmol·g^−1^, corresponding to a 51.2% increase compared with the control (Figure 3B; Table S3). In addition to superoxide anion accumulation, H_2_O_2_ levels in the soybean roots were also examined under SA stress. As shown in Figure 4A, the H_2_O_2_ content in CN26 increased from 0.99 ± 0.19 μmol·g^−1^ under control conditions to 2.54 ± 0.15 μmol·g^−1^ under the 21 mmol·L^−1^ Na^+^ treatment, and further increased significantly to 7.79 ± 0.27 μmol·g^−1^ under the 45 mmol·L^−1^ Na^+^ treatment, representing a 157% and 687% increase relative to the control, respectively. In JY441, the H_2_O_2_ content was 1.20 ± 0.11 μmol·g^−1^ in the control group, and increased progressively with rising stress intensity, reaching 1.56 ± 0.17 μmol·g^−1^ and 6.75 ± 0.34 μmol·g^−1^ under the 21 and 45 mmol·L^−1^ Na^+^ treatments, respectively, corresponding to increases of 30% and 463% relative to the control (Figure 4B).

Combined analysis of O_2_^−^ and H_2_O_2_ accumulation revealed distinct ROS profiles between the two cultivars. Under low-intensity stress (21 mmol·L^−1^ Na^+^), the O_2_^−^ content in CN26 increased sharply by 248% compared with the control (Figure 3A), whereas H_2_O_2_ increased by 157% (Figure 4A), indicating a significant imbalance between superoxide dismutase (SOD)-mediated O_2_^−^ dismutation and subsequent H_2_O_2_ detoxification. In contrast, JY441 exhibited a 25.2% decrease in the O_2_^−^ content under low-intensity stress (Figure 3B), accompanied by a 30% increase in H_2_O_2_ (Figure 4B), suggesting more efficient SOD activity that converts O_2_^−^ to H_2_O_2_ without excessive O_2_^−^ buildup. Under high-intensity stress (45 mmol·L^−1^ Na^+^), both the O_2_^−^ and H_2_O_2_ levels increased dramatically in both cultivars, indicating that severe stress overwhelms the antioxidant defense system. However, the fold increase in H_2_O_2_ was notably higher in CN26 (687%) than in JY441 (463%), suggesting a less coordinated ROS detoxification capacity in the sensitive cultivar. These results indicate that the differential regulation of ROS metabolism, particularly the balance between O_2_^−^ production and H_2_O_2_ accumulation, plays a critical role in determining cultivar-specific sensitivity to saline-alkaline stress.

#### 3.2.2. Effect of Saline-Alkaline Stress on POD Activity in Soybean Roots

**Figure 5:**
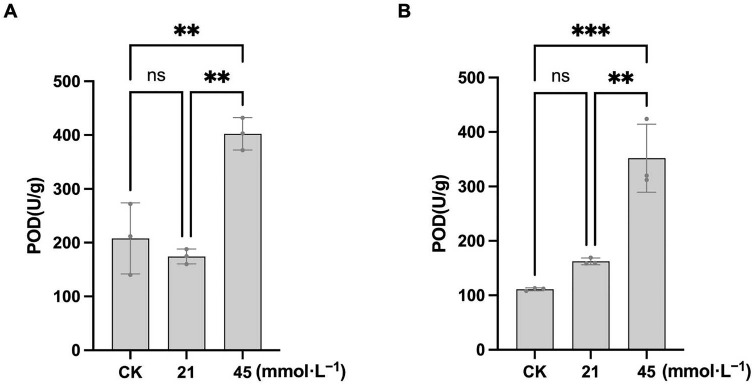
Saline-alkaline stress promotes the POD activity of soybean seedlings: (**A**) CN26. (**B**) JY 441. Data are presented as mean ± SD (n = 3 biological replicates), each replicate consisting of two individual plants with three technical replicates. ** and *** indicate significant differences at *p* < 0.01 and *p* < 0.001, respectively. CK, 0 mmol·L^−1^. ns, not significant.

During seed germination, the activity of POD in the soybean roots exhibited a complex concentration-dependent response to saline-alkaline stress. As shown in Figure 5A, the POD activity in CN26 under control conditions was 208.00 ± 66.09 U·g^−1^. At 21 mmol·L^−1^ Na^+^, the POD activity decreased slightly to 185.33 U·g^−1^ (10.9% reduction), with no significant difference from the control. Under the 45 mmol·L^−1^ Na^+^ treatment, the POD activity increased significantly to 372.00 U·g^−1^, representing a 78.8% increase compared with the control.

In JY 441, the POD activity in the control group was 173.33 U·g^−1^. At 21 mmol·L^−1^ Na^+^, the POD activity decreased significantly to 101.33 U·g^−1^ (41.5% reduction), suggesting that low-intensity stress may suppress the initial response of the plant antioxidant system. Under 45 mmol·L^−1^ Na^+^, the POD activity increased sharply to 518.67 U·g^−1^, corresponding to a 199% increase relative to the control and significantly higher than other treatments, indicating that severe stress activates the antioxidant defense system to mitigate enhanced oxidative damage (Figure 5B).

#### 3.2.3. Effect of Saline-Alkaline Stress on SOD Activity in Soybean Roots

**Figure 6:**
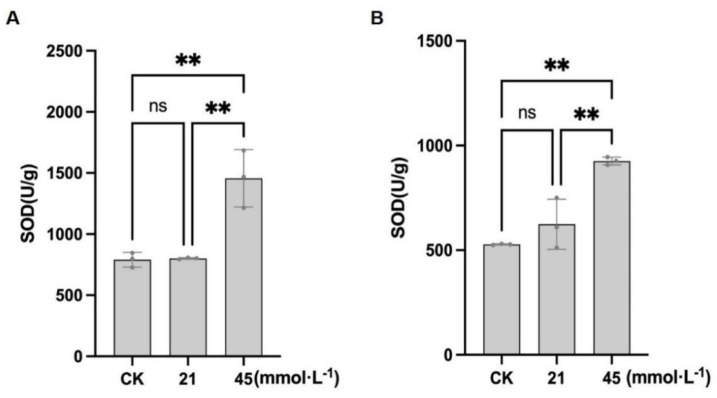
Effects of saline-alkaline stress on superoxide dismutase (SOD) activity in soybean seedlings: (**A**) CN26. (**B**) JY441. Data are presented as mean ± SD (n = 3 biological replicates), each replicate consisting of two individual plants with three technical replicates. ** indicates a significant difference at *p* < 0.01. CK, 0 mmol·L^−1^. ns, not significant.

Previous studies have reported that saline-alkaline stress increases SOD activity in soybean leaves. Although high-intensity stress markedly affects conventional soybean cultivars, hybrid cultivars are generally less impacted, with SOD activity being significantly enhanced under severe saline-alkaline stress, thereby improving the capacity to scavenge ROS [45]. SOD activity was further evaluated to assess antioxidant responses under saline-alkaline stress in the soybean roots. In CN26, the SOD activity under control conditions was 790.44 ± 59.96 U·mL^−1^. Treatment with 21 mmol·L^−1^ Na^+^ resulted in a slight increase to 801.25 ± 6.37 U·mL^−1^, with no significant difference from the control. Under 45 mmol·L^−1^ Na^+^, the SOD activity increased significantly to 1456.42 ± 234.73 U·mL^−1^, representing an 84.3% increase compared with the control (Figure 6A). In JY 441, the SOD activity under control conditions was 877.99 ± 264.78 U·mL^−1^. At 21 mmol·L^−1^ Na^+^, the SOD activity decreased to 590.52 ± 65.18 U·mL^−1^, with no significant difference from the control. Under the 45 mmol·L^−1^ Na^+^ treatment, the SOD activity increased to 859.75 ± 73.05 U·mL^−1^ (Figure 6B). Overall, the SOD activity was significantly elevated under high-intensity saline-alkaline stress in CN26, while JY 441 exhibited a recovery of SOD activity under severe stress conditions.

### 3.3. Saline-Alkaline Stress Induces DNA Damage in Soybean Seedling Roots

The preceding results demonstrated that saline-alkaline stress triggered excessive ROS accumulation in the soybean roots, potentially leading to genomic instability. To evaluate DNA integrity in seedling roots under saline-alkaline stress, a random amplified polymorphic DNA (RAPD) analysis was conducted to detect genome-wide polymorphic alterations. In RAPD assays, short arbitrary primers anneal to complementary sites within genomic DNA to amplify distinct fragments. Stress-induced sequence alterations may modify primer binding sites, resulting in the appearance or disappearance of RAPD bands. Because different abiotic stresses can differentially affect genomic DNA, primer selection is critical for ensuring analytical reliability [40].

**Figure 7:**
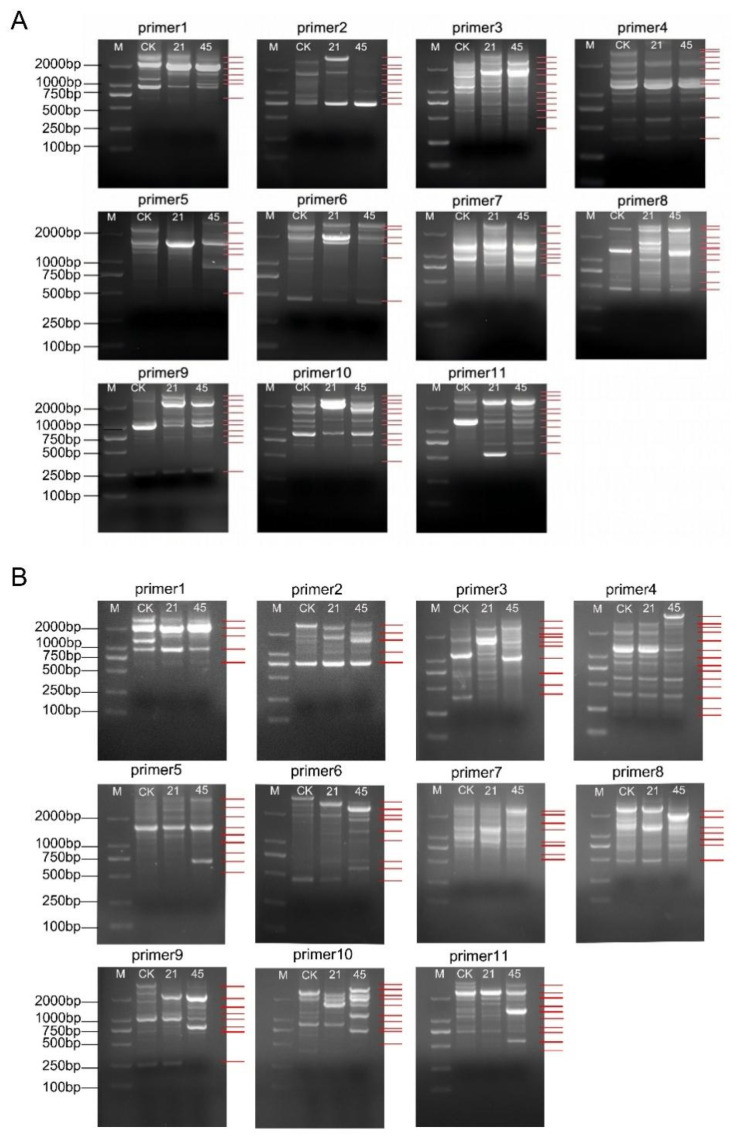
RAPD polymorphism analysis of genomic DNA in soybean seedling roots under saline-alkaline stress. Genomic DNA from roots treated with different Na^+^ concentrations (0, 21, and 45 mmol·L^−1^) was amplified using 11 random primers. (**A**) RAPD polymorphism patterns of CN26. (**B**) RAPD polymorphism patterns of JY441. M indicates the DL2000 DNA marker. Red arrows denote polymorphic bands relative to the CK. Bands were considered reproducible and included in polymorphism analysis when detected in at least two independent experimental replicates.

**Figure 8:**
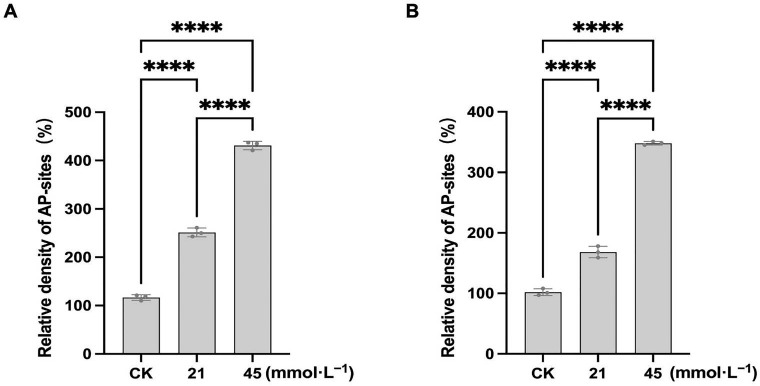
The relative density of AP sites in the total DNA of soybean seedling roots under saline-alkaline stress. (**A**) relative density of AP sites in the total DNA of CN26. (**B**) relative density of AP sites in the total DNA of JY441. Data are presented as mean ± SD (n = 3 biological replicates), each replicate consisting of three individual seedlings with three technical replicates. **** indicates significant differences at *p* < 0.0001.

As shown in Figure 7A, 11 selected primers amplified 77 RAPD fragments in the control samples of CN26. Under saline-alkaline stress at 21 and 45 mmol·L^−1^ Na^+^, the numbers of altered RAPD fragments were 71 and 61, respectively (Table S4). In JY441, 91 RAPD fragments were amplified under control conditions, whereas 48 and 68 altered fragments were detected under 21 and 45 mmol·L^−1^ treatments, respectively (Figure 7B, Table S5). Saline-alkaline stress reduced genomic template stability (GTS) in both cultivars. In JY441, GTS decreased to 47.25% and 25.27% under 21 and 45 mmol·L^−1^ treatments, respectively, whereas in CN26, GTS values were 7.79% and 20.78% under the corresponding treatments. To further evaluate the effect of saline-alkali stress on DNA oxidative damage in the roots of CN26 and JY441 seedlings, the relative density of apurinic/apyrimidinic (AP) sites in total DNA was measured. Saline-alkali stress significantly increased the relative density of AP sites in both cultivars. Notably, the number of AP sites in JY441 was lower than that in CN26 (Figure 8A,B). Collectively, these findings indicate that saline-alkaline stress induces substantial DNA damage in soybean seedling roots, with greater genomic instability observed under higher alkaline concentrations.

### 3.4. Effect of Saline-Alkaline Stress on DNA Damage Repair in Soybean Seedling Roots

To further elucidate the molecular response of soybean seedling roots to saline-alkaline stress, cell cycle progression and DNA damage repair-related gene expression were analyzed. Previous studies have demonstrated that ATM participates in the DNA damage response in Arabidopsis by repairing DNA double-strand breaks and delaying ROS-induced leaf senescence through stabilization of MKP2 phosphatase [46]. OsRAD51 plays a pivotal role in homologous recombination during meiosis and in the repair of DNA double-strand breaks in rice [47]. AtOGG1, a DNA glycosylase, enhances seed longevity and germination vigor under various stress conditions in Arabidopsis [48]. The transcription factor E2FA regulates cell cycle progression in G1/S checkpoints and serves as a critical link between nutrient signaling pathways, such as sugar–TOR signaling, and cell division [49]. In plants, WEE1 is transcriptionally activated upon DNA damage in an ATM-dependent manner and plays a key role in the intra-S and G2/M checkpoints, leading to cell cycle arrest to allow time for DNA repair [50,51]. In the present study, qRT-PCR was performed to determine the expression of five DNA damage repair-related genes: *ATM*, *OGG1*, *RAD4*, *RAD51*, *E2FA*, and *WEE1*.

**Figure 9:**
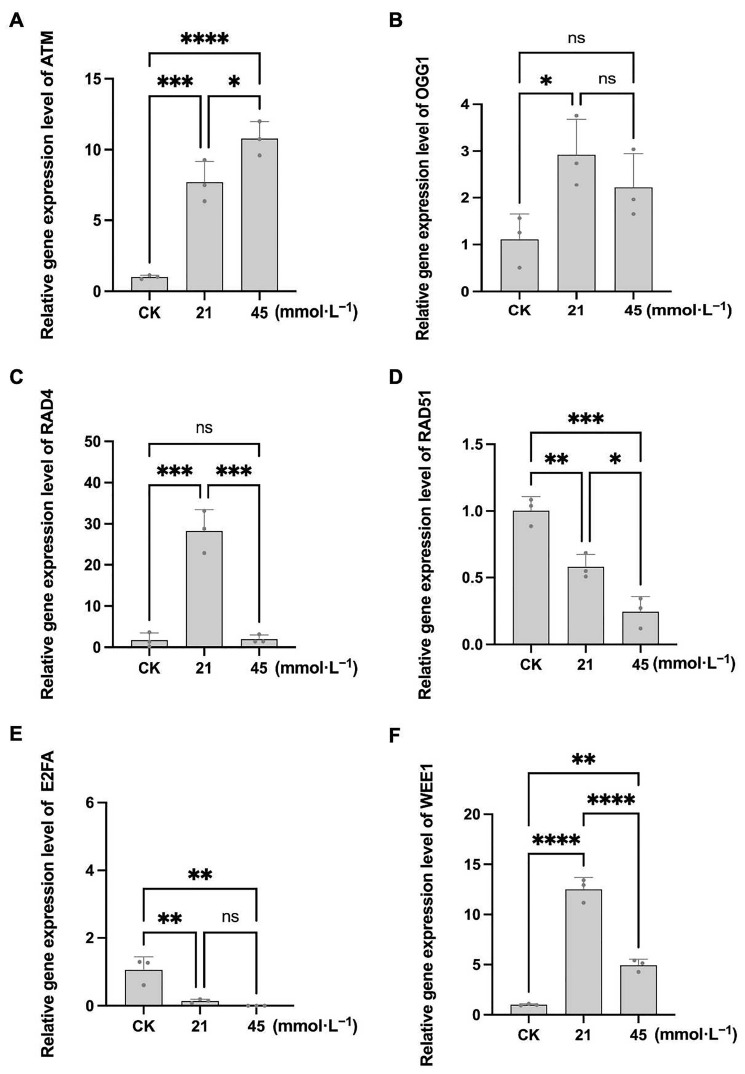
Relative expression levels of DNA damage repair-related genes in CN26 seedling roots under saline-alkaline stress. (**A**–**F**) Relative transcript levels of *ATM*, *OGG1*, *RAD4*, *RAD51*, *E2FA*, and WEE1, respectively. Data are presented as mean ± SD (n = 3 biological replicates), each replicate consisting of two individual plants with three technical replicates. *, **, ***, and **** indicate significant differences at *p* < 0.05, *p* < 0.01, *p* < 0.001, and *p* < 0.0001, respectively. CK, 0 mmol·L^−1^. ns, not significant.

**Figure 10:**
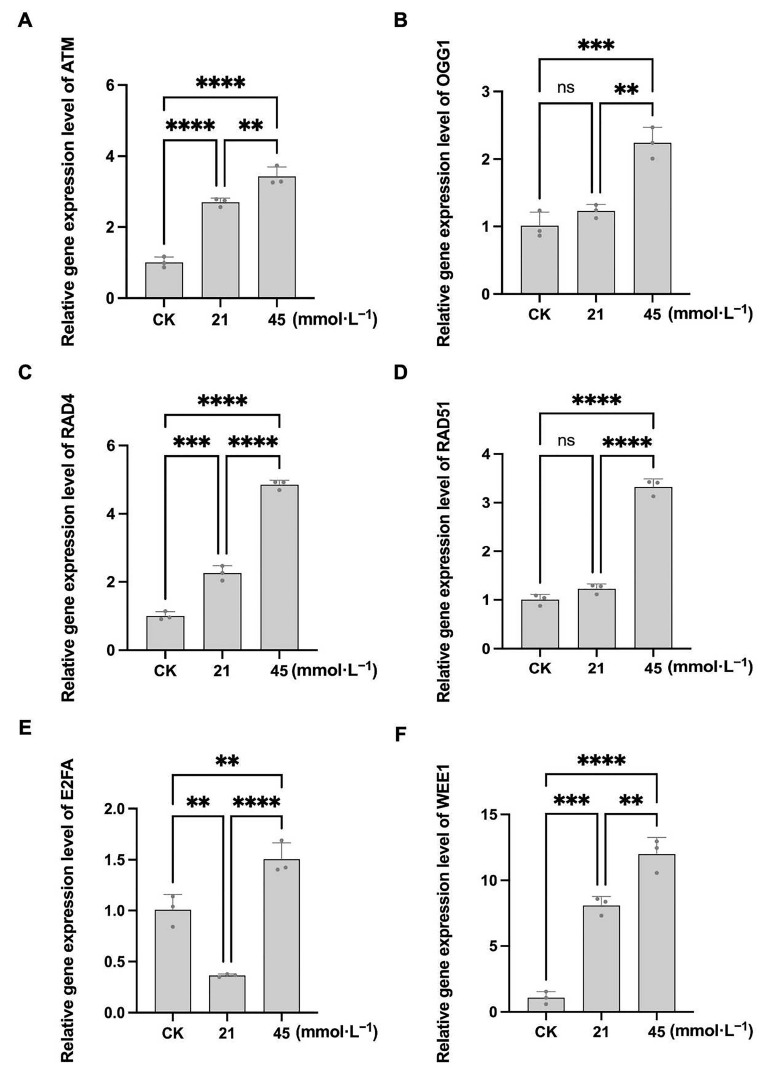
Relative expression levels of DNA damage repair-related genes in JY441 seedling roots under saline-alkaline stress. (**A**–**F**) Relative transcript levels of *ATM*, *OGG1*, *RAD4*, *RAD51*, *E2FA*, and WEE1, respectively. Data are presented as mean ± SD (n = 3 biological replicates), each replicate consisting of two individual plants with three technical replicates. **, ***, and **** indicate significant differences at *p* < 0.01, *p* < 0.001, and *p* < 0.0001, respectively. CK, 0 mmol·L^−1^. ns, not significant.

In CN26, compared with the control, the transcript levels of *ATM*, *OGG1*, and *RAD4* in germinating roots were significantly upregulated under 21 and 45 mmol·L^−1^ saline-alkaline stress, indicating activation of DNA repair pathways. However, under high-intensity stress, *OGG1* and *RAD4* expression decreased significantly. In contrast, *RAD51* and E2FA expression progressively declined with increasing stress intensity. Notably, WEE1 expression in CN26 was significantly induced under the 21 mmol·L^−1^ Na^+^ treatment (approximately 1.7-fold relative to control) but decreased under the 45 mmol·L^−1^ Na^+^ treatment (approximately 0.6-fold relative to control), suggesting that WEE1-mediated G2/M checkpoint activation occurs under stress conditions, but under severe stress this sensitive cultivar showed decreased G2/M checkpoint activation (Figure 9A–F). In JY441, the expression of ATM, OGG1, RAD51, and RAD4 increased markedly with rising saline-alkaline concentrations. Although OGG1 and RAD51 showed elevated expression under low-intensity stress, the differences were not statistically significant relative to the control. The expression of E2FA decreased under low-intensity stress but increased under high-intensity stress, indicating G1/S checkpoint activation. WEE1 expression in JY441 was strongly induced under low and high stress, suggesting that the G2/M checkpoint may be effectively activated in this tolerant cultivar (Figure 10A–F). Collectively, these results suggest that saline-alkaline stress induces substantial DNA damage in soybean seedling roots during germination. The sensitive cultivar CN26 initiates DNA repair and cell cycle arrest under mild stress but exhibits an attenuated repair response under severe stress. In contrast, the tolerant cultivar JY441 maintains a more robust DNA damage response under both low- and high-intensity saline-alkaline stress conditions.

## 4. Discussion

Saline-alkaline stress exerts multifaceted effects on soybean growth through the combined impacts of high pH, ionic toxicity, and osmotic imbalance. Previous studies have demonstrated that mixed alkaline stress at varying intensities significantly affects soybean development from germination to maturity, with phenotypic responses differing among cultivars according to their tolerance levels [9]. As stress severity increases, soybean growth is progressively inhibited, as reflected by reduced germination rates, impaired radicle elongation, and decreased biomass accumulation [52]. In the present study, saline-alkaline stress markedly reduced the germination rate and vigor of JY441, whereas its effect on CN26 germination was comparatively limited, with a slight increase in germination vigor observed under certain conditions. Low-dose stimulation of germination has been reported in some soybean cultivars and is generally attributed to hormesis, whereby mild stress activates protective mechanisms, including enhanced antioxidant enzyme activities and osmolyte accumulation [45]. Despite these differences during germination, saline-alkaline stress significantly suppressed root elongation in both cultivars, indicating that radicle growth is highly sensitive to alkaline stress.

Another intriguing observation from the phenotypic analysis (Figure 1) was that the tolerant cultivar JY441 retained the ability to form adventitious roots under low-intensity stress and exhibited less inhibition of hypocotyl elongation compared with CN26. However, hypocotyl thickness did not increase proportionally, suggesting a specific disruption of cytoskeletal or cell wall-related processes rather than maintenance of cell division. Previous studies have shown that hypocotyl thickening depends on cell wall biosynthesis and cell expansion, both of which require adequate water availability and turgor pressure [53,54]. Under saline-alkaline stress, reduced water uptake and osmotic imbalance may impair cell expansion and wall deposition, leading to restricted thickening even when elongation proceeds. The decline in the water content, particularly in both cultivars, may further limit cell expansion and wall deposition. Further studies are needed to clarify the specific mechanisms.

Excessive accumulation of ROS disrupts cellular homeostasis by oxidizing lipids, nucleic acids, and proteins, thereby impairing essential physiological processes [55]. In this study, saline-alkaline stress substantially increased hydrogen peroxide (H_2_O_2_) and superoxide anion (O_2_^−^) levels in the roots of both cultivars. Under low-intensity stress, ROS accumulation was lower in JY441 than in CN26, suggesting a stronger intrinsic tolerance in JY441. Collectively, these findings indicate that ROS overaccumulation is a primary factor contributing to SA-induced growth inhibition in soybean seedlings.

Antioxidant enzymes, including SOD, POD, CAT, APX, and GSH-related systems, play central roles in ROS detoxification [56]. In this study, saline-alkaline stress significantly enhanced SOD and POD activities in the soybean roots in a concentration-dependent manner, consistent with previous reports. Enzyme activities were generally higher in JY441 than in CN26. However, when ROS production exceeds the scavenging capacity of the antioxidant system, the system itself can be impaired, preventing further enhancement of enzyme activity and leading to plant damage [57]. Under low-intensity stress, delayed or insufficient activation of SOD and POD limited the efficiency of ROS removal. Moreover, the imbalance between SOD-mediated O_2_^−^ dismutation and POD-mediated H_2_O_2_ detoxification reduced overall antioxidant coordination, particularly in CN26. Under high-intensity stress, rapid ROS accumulation overwhelmed antioxidant defenses in both cultivars, resulting in pronounced inhibition of germination and root growth. These results suggest that soybean tolerance to saline-alkaline stress depends on maintaining a dynamic equilibrium between ROS production and scavenging capacity.

DNA represents a major intracellular target of ROS. Oxidative stress can induce base modifications, strand breaks, crosslinks, and chromosomal aberrations, thereby increasing genomic instability and cytotoxicity [15,16,58]. Persistent DNA damage may trigger programmed cell death and inhibit plant growth. In the present study, saline-alkaline stress significantly increased RAPD polymorphism, a widely accepted method for detecting genotoxic effects in plants, in soybean seedling roots, with greater polymorphic alterations detected in CN26 than in JY441, indicating more severe genomic instability in the sensitive cultivar. This observation may be associated with DNA damage tolerance (DDT), whereby cells temporarily tolerate certain lesions rather than immediately undergoing cell death [31,59]. These results are consistent with the observed expression patterns of DNA damage repair (DDR) and cell cycle-related genes (Figure 8 and Figure 9).

The DDR network comprises multiple repair pathways, including base excision repair (BER) and nucleotide excision repair (NER), which collectively mitigate oxidative DNA lesions [20,29]. In soybean seedling roots, saline-alkaline stress-induced oxidative DNA damage or amplified single-base oxidative lesions via DDT require repair [60,61]. ATM functions as a central regulator of the DNA damage response. OGG1 participates in the removal of oxidized bases, whereas RAD4 and RAD51 are essential components of NER and homologous recombination pathways, respectively. In this study, saline-alkaline stress significantly upregulated ATM, OGG1, RAD4, and RAD51 expression in JY441 roots but not consistently in CN26, highlighting cultivar-dependent differences in repair capacity. DNA damage sensors recognize lesions and activate checkpoints regulating cell cycle arrest, endoreplication, or apoptosis [62,63]. In animals, ATM/ATR signaling inhibits CDK2/CDK4 activity and mediates G1/S or G2/M arrest [64,65]. Zhao et al. [31] demonstrated that reduced expression of G1/S transition-related genes (e.g., *E2FA*) and G2/M transition-related genes (e.g., *WEE1*) is strongly associated with cell cycle arrest, as confirmed by flow cytometry. Similarly, Reichheld et al. [66] showed that oxidative stress impairs G1/S transition and delays mitotic entry, accompanied by repression of cell cycle gene expression. In the present study, saline-alkaline stress upregulated *ATM* in both cultivars, suggesting activation of G2/M checkpoint control. The concurrent downregulation of *E2FA* and upregulation of *WEE1* (Figure 9 and Figure 10) further indicate suppression of G1/S and G2/M transitions. Previous studies have shown that G2/M arrest affects cell division, while G1/S arrest inhibits DNA replication [31]. In CN26, decreased *E2FA* expression and increased *WEE1* expression indicate activation of both G1/S and G2/M checkpoints. In contrast, in JY441, *E2FA* expression decreased under low-intensity stress but increased under high-intensity stress, suggesting G1/S arrest under mild stress and partial recovery of DNA replication under severe stress. Meanwhile, *WEE1* was upregulated under both stress levels, indicating sustained G2/M checkpoint activation. These differences in checkpoint regulation may contribute to variation in saline-alkaline tolerance between cultivars. Although direct evidence of cell cycle arrest (e.g., flow cytometry) and cell division defects was not provided, the observed transcriptional changes in key regulators provide molecular evidence of altered cell cycle status. Future studies using cytological approaches are needed to directly assess cytoskeletal organization and chromosome dynamics under saline-alkaline stress. Overall, saline-alkaline stress exerts concentration-dependent and dynamic effects on soybean growth, involving coordinated physiological and molecular responses from antioxidant defense to genome maintenance and cell cycle regulation. These findings enhance our understanding of the mechanisms underlying alkaline tolerance and provide potential molecular targets for breeding stress-resilient soybean cultivars.

## 5. Conclusions

This study demonstrates that saline-alkaline stress inhibits soybean germination and early seedling growth through ROS overaccumulation, oxidative DNA damage, activation of DNA damage repair pathways, and modulation of cell cycle progression. Although saline-alkaline stress suppressed germination in both cultivars, JY441 exhibited a higher relative germination rate and vigor than CN26, indicating superior intrinsic tolerance during the germination stage. Compared with CN26, JY441 more effectively activated antioxidant defenses, cell cycle checkpoints, and DNA repair pathways under saline-alkaline stress, thereby mitigating ROS-induced genomic damage. These results provide mechanistic insights into soybean responses to alkaline stress and identify potential physiological and molecular indicators for breeding cultivars with enhanced tolerance, particularly during early developmental stages.

## Figures and Tables

**Table 1:** Effects of saline-alkaline stress on the germination parameters of soybean.

	Na^+^ Concentration (mmol·L^−1^)	Germination Potential (%)	Germination Rate (%)	Root Length (cm)	Fresh Weight (g)	Dry Weight (g)
CN26	0	0.22 ± 0.11 a	0.34 ± 0.19 a	16.30 ± 2.33 a	1.06 ± 0.15 a	0.2 ± 0.01 a
	21	0.34 ± 0.15 a	0.36 ± 0.15 a	9.41 ± 1.06 b	0.79 ± 0.07 b	0.2 ± 0.01 a
	45	0.32 ± 0.16 a	0.38 ± 0.15 a	3.07 ± 0.17 b	0.76 ± 0.07 b	0.21 ± 0.02 a
JY441	0	0.96 ± 0.04 a	0.98 ± 0.02 a	13.94 ± 3.10 a	1.07 ± 0.06 a	0.17 ± 0.02 a
	21	0.74 ± 0.06 b	0.94 ± 0.07 b	10.08 ± 3.03 a	0.75 ± 0.05 b	0.15 ± 0.01 ab
	45	0.40 ± 0.02 b	0.80 ± 0.07 b	3.48 ± 0.84 b	0.54 ± 0.04 c	0.14 ± 0.02 b

Note: Data are expressed as means ± SD. n = 3 replicates, each consisting of an average of 10 individual plants. For the same cultivar, different letters indicate statistically significant differences (*p* < 0.05) among the treatments.

## Data Availability

The original contributions presented in this study are included in the article/Supplementary Material. Further inquiries can be directed to the corresponding author.

## Supplementary Materials

The following supporting information can be downloaded at https://www.mdpi.com/article/10.3390/plants15071131/s1. Table S1. RAPD random primers. Table S2. qPCR primers. Table S3. Physiological parameters of Chang 26 and JY 441 under saline-alkaline stress. Table S4. Statistical analysis of RAPD polymorphism of genomic DNA in the roots of Chang 26 seedlings under saline-alkaline stress. Table S5. Statistical analysis of RAPD polymorphism of genomic DNA in the roots of JY 441 seedlings under saline-alkaline stress.

## References

[1] Robles P., Quesada V. (2019). Transcriptional and Post-transcriptional Regulation of Organellar Gene Expression (OGE) and Its Roles in Plant Salt Tolerance. Int. J. Mol. Sci..

[2] Yang Y., Guo Y. (2018). Unraveling salt stress signaling in plants. J. Integr. Plant Biol..

[3] Liu J., Shen F., Xiao Y., Fang H., Han Z. (2020). Genomics-assisted prediction of salt and alkali tolerances and functional marker development in apple rootstocks. BMC Genom..

[4] Zhang X., Ma X., Wang S., Liu S., Shi S. (2024). Physiological and Genetic Aspects of Resistance to Abiotic Stresses in Capsicum Species. Plants.

[5] Sakamoto A., Murata A.N. (1998). Metabolic engineering of rice leading to biosynthesis of glycinebetaine and tolerance to salt and cold. Plant Mol. Biol..

[6] van Zelm E., Zhang Y., Testerink C. (2020). Salt Tolerance Mechanisms of Plants. Annu. Rev. Plant Biol..

[7] Fan X.D., Wang J.Q., Yang N., Dong Y.Y., Liu L., Wang F.W., Wang N., Chen H., Liu W.C., Sun Y.P. (2013). Gene expression profiling of soybean leaves and roots under salt, saline-alkali and drought stress by high-throughput Illumina sequencing. Gene.

[8] Dai L.-Y., Zhu H.-D., Yin K.-D., Du J.-D., Zhang Y.-X. (2017). Seed priming mitigates the effects of saline-alkali stress in soybean seedlings. Chil. J. Agric. Res..

[9] Li S., Xu L., Li Y., Waqar A., Hu Z., Yang M., Zhao Y., Qi Z., Chen Q., Hu L. (2025). Advances in Salinity Tolerance of Soybean: Molecular Mechanism and Breeding Strategy. Food Energy Secur..

[10] Zhao Y., Wang G., Zhao M., Wang M., Jiang M. (2021). Direct and indirect effects of soil salinization on soil seed banks in salinizing wetlands in the Songnen Plain, China. Sci. Total Environ..

[11] Yuan Y., Zu M., Li R., Zuo J., Tao J. (2023). Soil properties, microbial diversity, and changes in the functionality of saline-alkali soil are driven by microplastics. J. Hazard. Mater..

[12] Rao Y., Peng T., Xue S. (2023). Mechanisms of plant saline-alkaline tolerance. J. Plant Physiol..

[13] Sharma M., Tisarum R., Kohli R.K., Batish D.R., Cha-Um S., Singh H.P. (2024). Inroads into saline-alkaline stress response in plants: Unravelling morphological, physiological, biochemical, and molecular mechanisms. Planta.

[14] Ge Y., Li Y., Zhu Y.M., Bai X., Lv D.K., Guo D., Ji W., Cai H. (2010). Global transcriptome profiling of wild soybean (*Glycine soja*) roots under NaHCO_3_ treatment. BMC Plant Biol..

[15] Dmitrieva N.I., Cai Q., Burg M.B. (2004). Cells adapted to high NaCl have many DNA breaks and impaired DNA repair both in cell culture and in vivo. Proc. Natl. Acad. Sci. USA.

[16] Saha P., Mukherjee A., Biswas A.K. (2015). Modulation of NaCl induced DNA damage and oxidative stress in mungbean by pretreatment with sublethal dose. Biol. Plant..

[17] He J., Lin J., Wang N., Yang J., Yang X., Chen Y., Zhang M., Chen R., Chen X., Dong L. (2025). The clock component LHY1b negatively regulates alkaline stress by repressing AOX1-mediated oxidative responses in soybean. Plant Physiol..

[18] Chmielowska-Bąk J., Izbiańska K., Ekner-Grzyb A., Bayar M., Deckert J. (2017). Cadmium Stress Leads to Rapid Increase in RNA Oxidative Modifications in Soybean Seedlings. Front. Plant Sci..

[19] Chmielowska-Bąk J., Deckert J. (2012). A common response to common danger? Comparison of animal and plant signaling pathways involved in cadmium sensing. J. Cell Commun. Signal..

[20] Scott T.L., Rangaswamy S., Wicker C.A., Izumi T. (2014). Repair of oxidative DNA damage and cancer: Recent progress in DNA base excision repair. Antioxid. Redox Signal..

[21] Wei H., Movahedi A., Xu C., Sun W., Wang P., Li D., Yin T., Zhuge Q. (2020). Characterization, Expression Profiling, and Functional Analysis of PtDef, a Defensin-Encoding Gene From Populus trichocarpa. Front. Microbiol..

[22] Sobkowiak R., Rymer K., Rucińska R., Deckert J. (2004). Cadmium-induced changes in antioxidant enzymes in suspension culture of soybean cells. Acta Biochim. Pol..

[23] Yang W., Wang F., Liu L.N., Sui N. (2020). Responses of Membranes and the Photosynthetic Apparatus to Salt Stress in Cyanobacteria. Front. Plant Sci..

[24] Bian X., He J., Li B., Ma X., Meng Y., Shang X., Si E., Wang H., Wang J., Yang K. (2020). Effects of drought and salt stress on seed germination characteristics of Halogeton glomeratus. Acta Prataculturae Sin..

[25] Zhang Y., Liu S., Liang X., Zheng J., Lu X., Zhao J., Li H., Zhan Y., Teng W., Li H. (2025). GmFER1, a soybean ferritin, enhances tolerance to salt stress and root rot disease and improves soybean yield. Plant Biotechnol. J..

[26] Dong Z., Huang J., Qi T., Fu Q., Meng A., Fu Y. (2023). Effects of Plant Regulators on the Seed Germination and Antioxidant Enzyme Activity of Cotton under Compound Salt Stress. Plants.

[27] Roldán-Arjona T., Ariza R.R. (2009). Repair and tolerance of oxidative DNA damage in plants. Mutat. Res..

[28] Berquist B.R., Wilson D.M. (2012). Pathways for repairing and tolerating the spectrum of oxidative DNA lesions. Cancer Lett..

[29] Melis J.P., van Steeg H., Luijten M. (2013). Oxidative DNA damage and nucleotide excision repair. Antioxid. Redox Signal..

[30] Slupphaug G., Kavli B., Krokan H.E. (2003). The interacting pathways for prevention and repair of oxidative DNA damage. Mutat. Res..

[31] Zhao Q., Wang H., Du Y., Rogers H.J., Wu Z., Jia S., Yao X., Xie F., Liu W. (2020). MSH2 and MSH6 in Mismatch Repair System Account for Soybean (*Glycine max* (L.) Merr.) Tolerance to Cadmium Toxicity by Determining DNA Damage Response. J. Agric. Food Chem..

[32] Wang Z., Li Z., Wang Z., Liu T., Zhang P., Li S., Ye S., Yang K., Gai Z., Liu L. (2025). Alkaline stress suppresses soybean waterlogging tolerance by exacerbating energy expenditure and ROS accumulation. Plant Physiol. Biochem..

[33] Ni X., Wang Y., Dai L., Jiang K., Zeng S., Huang Y., Zhou Y., Duan L., Bian C., Liu Q. (2025). The transcription factor GmbZIP131 enhances soybean salt tolerance by regulating flavonoid biosynthesis. Plant Physiol..

[34] Ashraf M., Foolad M.R. (2007). Roles of glycine betaine and proline in improving plant abiotic stress resistance. Environ. Exp. Bot..

[35] Ding W., Lin J., Li C., Zhu Z., Wu C., Cao J., Liu D., Zhang Y., Yang Q., Xing A. (2025). Development of a comprehensive evaluation system and models to determine soybean seed vigor. Ind. Crop. Prod..

[36] Giannopolitis C.N., Ries S.K. (1977). Superoxide dismutases: I. Occurrence in higher plants. Plant Physiol..

[37] Li H.-X., Xiao Y., Cao L.-L., Yan X., Li C., Shi H.-Y., Wang J.-W., Ye Y.-H. (2013). Cerebroside C increases tolerance to chilling injury and alters lipid composition in wheat roots. PLoS ONE.

[38] Elstner E.F., Heupel A. (1976). Inhibition of nitrite formation from hydroxylammoniumchloride: A simple assay for superoxide dismutase. Anal. Biochem..

[39] Velikova V., Yordanov I., Edreva A. (2000). Oxidative stress and some antioxidant systems in acid rain-treated bean plants: Protective role of exogenous polyamines. Plant Sci..

[40] Chen S., Zhao Q., Yu G., Ren C., Zhang Y. (2022). DNA damage of roots in soybean seedlings under salt stress detected by random amplified polymorphic (RAPD) analysis. Chin. J. Ecol..

[41] Wang H., He L., Song J., Cui W., Zhang Y., Jia C., Francis D., Rogers H.J., Sun L., Tai P. (2016). Cadmium-induced genomic instability in Arabidopsis: Molecular toxicological biomarkers for early diagnosis of cadmium stress. Chemosphere.

[42] Livak K.J., Schmittgen T.D. (2001). Analysis of relative gene expression data using real-time quantitative PCR and the 2(-Delta Delta C(T)) Method. Methods.

[43] Chi X., Zhang Y., Li Z. (2020). Effects of alkaline stress on photosynthetic characteristics and endogenous hormone contents of soybean. Jiangsu J. Agric. Sci..

[44] Wang G., Shen W., Zhang Z., Guo S., Hu J., Feng R., Zhao Q., Du J., Du Y. (2022). The Effect of Neutral Salt and Alkaline Stress with the Same Na^+^ Concentration on Root Growth of Soybean (*Glycine max* (L.) Merr.) Seedlings. Agronomy.

[45] Qiu S., Zhang Y., Sun H., Liu L., Li C., Hua Z., Dong H. (2025). Integrated transcriptomics and metabolomics elucidate additive inhibitory effects of combined salinity-waterlogging stress on soybean growth and metabolic adaptations. Plant Physiol. Biochem..

[46] Pedroza-Garcia J.A., Eekhout T., Achon I., Nisa M.-U., Coussens G., Vercauteren I., Van den Daele H., Pauwels L., Van Lijsebettens M., Raynaud C. (2021). Maize ATR safeguards genome stability during kernel development to prevent early endosperm endocycle onset and cell death. Plant Cell.

[47] Liu X., Cao Y., Du G., Zhang C., Xu M., Cheng Z., Shen Y., Yu H. (2022). OsRAD51 Plays a Vital Role in Promoting Homologous Recombination in Rice Meiosis. Int. J. Mol. Sci..

[48] Chen H., Chu P., Zhou Y., Li Y., Liu J., Ding Y., Tsang E.W., Jiang L., Wu K., Huang S. (2012). Overexpression of AtOGG1, a DNA glycosylase/AP lyase, enhances seed longevity and abiotic stress tolerance in Arabidopsis. J. Exp. Bot..

[49] Nisa M., Eekhout T., Bergis C., Pedroza-Garcia J.-A., He X., Mazubert C., Vercauteren I., Cools T., Brik-Chaouche R., Drouin-Wahbi J. (2023). Distinctive and complementary roles of E2F transcription factors during plant replication stress responses. Mol. Plant.

[50] Pedroza-Garcia J.A., Xiang Y., De Veylder L. (2022). Cell cycle checkpoint control in response to DNA damage by environmental stresses. Plant J..

[51] De Schutter K., Joubès J., Cools T., Verkest A., Corellou F., Babiychuk E., Van Der Schueren E., Beeckman T., Kushnir S., Inzé D. (2007). Arabidopsis WEE1 kinase controls cell cycle arrest in response to activation of the DNA integrity checkpoint. Plant Cell.

[52] Atienzar F.A., Conradi M., Evenden A.J., Jha A.N., Depledge M.H. (1999). Qualitative assessment of genotoxicity using random amplified polymorphic DNA: Comparison of genomic template stability with key fitness parameters in Daphnia magna exposed to benzo[a]pyrene. Environ. Toxicol. Chem..

[53] Meyer R.F., Boyer J.S. (1972). Sensitivity of cell division and cell elongation to low water potentials in soybean hypocotyls. Planta.

[54] Wang C., Chen Y., Cui C., Shan F., Zhang R., Lyu X., Lyu L., Chang H., Yan C., Ma C. (2023). Blue Light Regulates Cell Wall Structure and Carbohydrate Metabolism of Soybean Hypocotyl. Int. J. Mol. Sci..

[55] Zhao X., Fang J., Li S., Gaur U., Xing X., Wang H., Zheng W. (2019). Artemisinin Attenuated Hydrogen Peroxide (H2O2)-Induced Oxidative Injury in SH-SY5Y and Hippocampal Neurons via the Activation of AMPK Pathway. Int. J. Mol. Sci..

[56] Mujahid A., Muhammad H.M.D. (2024). Insights into Different Mitigation Approaches for Abiotic Stress in Horticultural Plants. Adv. Plant Sci. Environ..

[57] Meloni D.A., Oliva M.A., Martinez C.A., Cambraia J. (2003). Photosynthesis and activity of superoxide dismutase, peroxidase and glutathione reductase in cotton under salt stress. Environ. Exp. Bot..

[58] Asik E., Kashif S.Z., Oztas O. (2025). Plant tolerance mechanisms to DNA-damaging UV stress. J. Exp. Bot..

[59] Andersen P.L., Xu F., Xiao W. (2008). Eukaryotic DNA damage tolerance and translesion synthesis through covalent modifications of PCNA. Cell Res..

[60] Chang D.J., Cimprich K.A. (2009). DNA damage tolerance: When it’s OK to make mistakes. Nat. Chem. Biol..

[61] Waters L.S., Minesinger B.K., Wiltrout M.E., D’Souza S., Woodruff R.V., Walker G.C. (2009). Eukaryotic translesion polymerases and their roles and regulation in DNA damage tolerance. Microbiol. Mol. Biol. Rev..

[62] Hartwell L.H., Kastan M.B. (1994). Cell cycle control and cancer. Science.

[63] Seifert M., Scherer S.J., Edelmann W., Böhm M., Meineke V., Löbrich M., Tilgen W., Reichrath J. (2008). The DNA-mismatch repair enzyme hMSH2 modulates UV-B-induced cell cycle arrest and apoptosis in melanoma cells. J. Investig. Dermatol..

[64] Cools T., De Veylder L. (2009). DNA stress checkpoint control and plant development. Curr. Opin. Plant Biol..

[65] Spampinato C.P. (2017). Protecting DNA from errors and damage: An overview of DNA repair mechanisms in plants compared to mammals. Cell. Mol. Life Sci..

[66] Reichheld J.-P., Vernoux T., Lardon F., Van Montagu M., Inzé D. (1999). Specific checkpoints regulate plant cell cycle progression in response to oxidative stress. Plant J..

