# Supplementary material for: Saline-Alkaline Stress Suppresses Soybean Germination and Early Seedling Growth via Induction of DNA Damage in Roots
Source: Plants (Basel). 2026 Apr 7;15(7):1131. doi: 10.3390/plants15071131 (PMC13075964; doi:10.3390/plants15071131)
Supplement: Supplementary file 1 [file plants-15-01131-s001.zip › plants-4220863-supplementary.pdf]

Table S1. RAPD random primers

Primer number	Primer sequence	GC (%)	Tm (℃)
Random primer 1	CTGGCGAACT	55	32
Random primer 2	TCCGATGCTG	55	32
Random primer 3	CTGCGCTGGA	64	34
Random primer 4	CTGAGGTCTC	55	32
Random primer 5	CTGGGGCTGA	64	34
Random primer 6	TCTCCGCCCT	64	34
Random primer 7	GGTGGTGATG	55	32
Random primer 8	AAAGTGCGGC	55	32
Random primer 9	ACCTTTGCGG	55	32
Random primer 10	AGACCCAGAG	55	32
Random primer 11	AATGCGGGAG	55	32

Table S2 qPCR primers

Primer name	Forward primer (5'-3')	Reverse primer (5'-3')
Tubulin	AGGTCGGAAACTCCTGCTGG	AAGGTGTTGAAGGCGTCGTG
RAD51	AGTAGATGGTTCTGCAGTCTTTGC	CTTTCCTGAGAGCTAGCCTCGTTG
OGG1	TGGGTCCTAAAGTGGCTGCTTG	ACGTGTGTGTCAACAGGAACAGC
RAD4	ATGGGAAGTGGCAACTGGAACC	ACTTGACCTCGCTCGTTCTTGG
E2Fa	ATGACGGAAATGGTTCCTGCT	TCAGAGAATGCATGAGAAGAGAGC
ATM	ACGTGCACTTCTCCGTGTCAAG	CCATGGATGCTTCGCATTTCGC

Table S3. Physiological parameters of Chang 26 and JY 441 under saline–alkaline stress

	Na^+ concetration （$mmol·L^{-1}$）	POD activity （U/g）	SOD activity （U/g）	Superoxide anion content （nmol/g）	H_2O_2 content （umol/g）
Chang 26	0	208.00±66.09b	790.44±59.96b	25.09±6.75b	1.2±0.11c
	21	185.33±24.11b	801.25±6.37b	87.37±13.39a	1.56±0.17b
	45	372.00±60.00a	1456.42±234.73a	108.51±4.85a	6.75±0.34a
JY441	0	173.33±67.76b	877.99±264.78a	132.49±26.01c	0.99±0.19b
	21	101.33±18.67b	590.52±65.18b	99.07±19.94b	2.54±0.16b
	45	518.67±67.72a	859.75±73.05a	200.32±38.08a	7.79±0.27a

Notes: Different lowercase letters indicate significant differences at the 0.05 probability level ($p<0.05$). The data are presented as means ± standard deviation (SD) calculated from three repetitions.

Table S4. Statistical analysis of RAPD polymorphism of genomic DNA in the roots of Chang 26 seedlings under saline–alkaline stress

NO.of primers	The Statistic of RAPD morphism（bp）				
	0 mmol·L^{-1} Na^{+}	21 mmol·L^{-1} Na^{+}		45 mmol·L^{-1} Na^{+}	
		+	-	+	-
Primer 1	5	5	3	5	4
Primer 2	5	4	4	4	3
Primer 3	10	6	4	6	4
Primer 4	10	1	2	0	1
Primer 5	6	1	2	3	3
Primer 6	5	4	3	2	1
Primer 7	4	4	1	2	1
Primer 8	10	6	4	7	3
Primer 9	6	4	1	3	1
Primer 10	8	4	1	1	1
Primer 11	8	5	2	5	3
TOTAL	77	44	27	36	25
	77	71		61	

Table S5. Statistical analysis of RAPD polymorphism of genomic DNA in the roots of JY 441 seedlings under saline–alkaline stress

NO.of primers	The Statistic of RAPD morphism（bp）				
	0 mmol·L^{-1} Na^{+}	21 mmol·L^{-1} Na^{+}		45 mmol·L^{-1} Na^{+}	
		+	-	+	-
Primer 1	6	1	1	1	1
Primer 2	6	1	1	1	3
Primer 3	10	6	4	7	5
Primer 4	11	0	1	3	1
Primer 5	8	4	2	3	1
Primer 6	6	2	2	6	5
Primer 7	6	2	1	1	2
Primer 8	9	1	1	2	1
Primer 9	11	1	5	4	4
Primer 10	8	2	4	4	5
Primer 11	10	3	4	5	3
TOTAL	91	23	25	37	31
	91	48		68	
